# Associations between diabetic retinopathy, mortality, disease, and mental health: an umbrella review of observational meta-analyses

**DOI:** 10.1186/s12902-022-01236-8

**Published:** 2022-12-09

**Authors:** Mike Trott, Robin Driscoll, Shahina Pardhan

**Affiliations:** 1 Vision and Eye Research Institute (VERI), Anglia Ruskin University, Young Street, Cambridge, CB1 2LZ UK; 2 Centre for Public Health, Queen’s University Belfast, Belfast, UK

**Keywords:** Umbrella review, Diabetic retinopathy, Mortality

## Abstract

**Background:**

Diabetic retinopathy is a complication of diabetes affecting the eyes and can lead to blindless if left untreated. Several significant risk factors have been reported for DR, of which several can be classified as some form of disease. Furthermore, several systematic reviews have reported associations between several types of mortality and DR. Numerous meta-analyses have pooled the data on these factors, however, a systematic evaluation of these meta-analytic relationships is lacking. In this study, therefore, we performed an umbrella review of systematic reviews of meta-analyses for mortality, diseases and DR, grading the credibility of evidence.

**Methods:**

A comprehensive database search for observational meta-analyses was conducted from inception until 29/04/2022 against pre-published inclusion criteria. For each meta-analytic outcome, a random-effects meta-analysis was re-conducted, stratifying by study design (and type of DR where possible) of included studies. Several statistical variables, including publication bias, heterogeneity, excess significance bias, and prediction intervals were used to grade the credibility of significant evidence from I to IV, using the recommendations from the Grading of Recommendations, Assessment, Development, and Evaluation (GRADE) criteria.

**Results:**

Of the 1,834 initial results, 11 systematic reviews with meta-analyses were included covering 16 independent outcomes (total participants = 299,655; median participants per outcome: 7,266; median individual studies per outcome = 5). Overall, 10/16 outcomes (62.5%) yielded significant results, most of which were graded as ‘highly suggestive’ (Grade II) evidence. DR was associated with all-cause and cardio-vascular mortality, obstructive sleep apnoea, depression eating disorders, and several forms of cognitive impairment.

**Conclusions:**

Results show highly suggestive evidence for associations between health outcomes and/or conditions and DR. Public health professionals and practitioners should note these findings when developing and/or reviewing public health polices.

**Supplementary Information:**

The online version contains supplementary material available at 10.1186/s12902-022-01236-8.

## Background

Diabetic retinopathy (DR) can be characterised as a microvascular complication of diabetes, with microvascular changes causing bleeding into the eye, which can cause visual impairment and blindness if treatment is not administered in a timely fashion [1]. It is also the leading cause of blindness among adults with diabetes [2]. DR can be characterised in several ways, including background retinopathy (none or some bleeding into the eye, not usually affecting vision), pre-proliferative retinopathy (more significant bleeding into the eye, which can affect vision), proliferative retinopathy (the appearance of scar tissue and new blood vessels, with some vision loss), and macular oedema (DME) [3]. Global prevalence of DR has been reported as being as high as 22% in people with diabetes, with the burden of the condition projected to worsen through to 2045 [4].

Several systematic reviews with accompanying meta-analyses have been conducted examining associations between DR and wide range of conditions, including different types of mortality [5, 6], mental health conditions [7], and other diseases [8] not typically associated with diabetes (e.g. obstructive sleep apnoea). To date, the epidemiolocal credibility of these associations have not been assessed.

In order to address the breadth of meta-analytic literature across multiple outcomes in conditions such as DR, studies re increasingly adopting reviews of reviews (commonly called umbrella reviews), which can use novel evidence synthesis strategies to capture the breadth of outcomes associated with a given exposure [9, 10]. For example, a recent umbrella review found that several modifiable risk factors were associated with DR, including vitamin D status and physical activity [11].

The aim of this study was to examine the strength and credibility of evidence on the associations between DR and mortality, mental health condition, or other disease (that is not a diabetic complication), derived from published meta-analyses of existing observational studies using a robust, systematic umbrella review approach.

The results of this study will inform practitioners, patients, and public health policy makers as to the quality/credibility of existing evidence in order to target interventions, inform public health policy, and also to inform further research.

## Methods

An umbrella review was performed, which adhered to the Preferred Reporting Items for Systematic Reviews and Meta-analyses (PRISMA) recommendations [12] and the Meta-analysis of Observational Studies in Epidemiology (MOOSE) guidelines [13]. The protocol was pre-published in the International prospective register of systematic reviews (PROSPERO registration number CRD42021245162).

### Search strategy and selection criteria

The following databases were searched: Embase, Pubmed, and CINAHL from inception to 29/04/2022. Key terms relating to DR and systematic reviews were used in the search (full search information can be found in Supplementary Table 1). Two independent reviewers searched titles/abstracts for eligibility against the following inclusion criteria:

1. Study design: systematic reviews with meta-analyses pooling observational (cross-sectional, case–control, cohort) studies
2. Examining relationships between DR and/or DME and any disease, mental health condition, or mortality.

Exclusion criteria were:

1. Studies published in languages other than English, French, Italian, or Spanish
2. Studies examining associations between DR and diabetic complications (e.g. neuropathy and diabetic kidney disease);
3. Meta-analyses that did not include individual study level effect sizes.

After title and abstract screening, the full text articles of remaining articles were retriaved and assessed by two independent reviewers. A third reviewer mediated any potential conflict and made a final decision where a consensus was not reached. When more than one meta-analysis assessed the same type of mortality, mental health condition, or disease, the meta-analysis with the largest *k* studies was retained, in line with methodology used in previous umbrella reviews [14–16].

### Data extraction

Two investigators (MT,RD) independently extracted data into a bespoke spreadsheet with the following information: PMID/DOI; author details; year; study design; population demographics; *k* included studies; total sample size (*n*); individual study effect sizes and 95% confidence intervals (CIs).

The methodological quality of each included meta-analysis was assessed with the Assessment of multiple systematic reviews (AMSTAR) 2 tool [17] independently by the same two investigators.

### Data analysis

For each individual study data, the meta-analysis was re-performed, calculating the pooled effect size with 95% CIs using a random-effects model, stratified by study design ( case–control/cross-sectional, or cohort [18]). If included meta-analyses stratified outcomes according to the different types of DR, these stratified analyses were also re-preformed. Heterogeneity was assessed with the I^2^ statistic, with < 50% being considered low, 50–75% being considered moderate, and > 75% being considered high [19]. Additionally, 95% prediction intervals (PIs) were calculated to determine whether or not the effect size could be appropriately applied to a population [20]. The presence of small-study effect bias was also tested, which was deemed to be present in case of (a) the pooled estimate being larger than the effect size of the largest study (defined as having the smallest standard error), and (b) the presence of publication bias (Egger’s regression asymmetry test *p* < 0.10) [14, 21–23]. Furthermore, the excess significance bias test was conducted by evaluating whether the observed number of significant studies was statistically different from the expected number of studies with statistically significant results (significance threshold set at *p* < 0.10) [23, 24], a test designed to assess whether the published meta-analyses comprise an over-representation of false positive findings [23]*.*

### Assessment of the credibility of the evidence

The credibility of analyses was assessed according to stringent criteria based on previously published umbrella reviews [11, 16, 25–27]. In brief, significant pooled effect sizes from the re-performed meta-analysis were graded as Grade I, II, III, or IV based on several stringent criteria, including the strength of the effect size (including the PI excluding the null), the presence of small study effects, and the presence of excess significance bias (full criteria available in Table 1).

**Table 1:** Credibility assessment criteria and grading

Grading of evidence	Criteria
Grade I^*^	- Statistical significance of *p* < 1*10^–6^, including more than 1,000 cases (or more than 20, 000 participants for continuous outcomes) - Have the largest component study reporting a significant result (*p* < 0.05), have a 95% prediction interval that excluded the null - Did not have large heterogeneity (I^2^ < 50%) - Showed no evidence of small study effects (*p* > 0.10) and excess significance bias (*p* > 0.10)
Grade II^*^	- Significance of *p* < 0.001, including more than 1,000 cases (or more than 20,000 participants for continuous outcomes) - Have the largest component study reporting a statistically significant result (*p* < 0.05)
Grade III^*^	- Significance of *p* < 0.01 with more than 1,000 cases (or more than 20,000 participants for continuous outcomes)
Grade IV	- Remaining significant associations with *p* < 0.05

^*^Note that an AMSTAR grade of ‘low’ or ‘critically low’ downgraded the classification by one grade

## Results

### Search

**Fig. 1:**
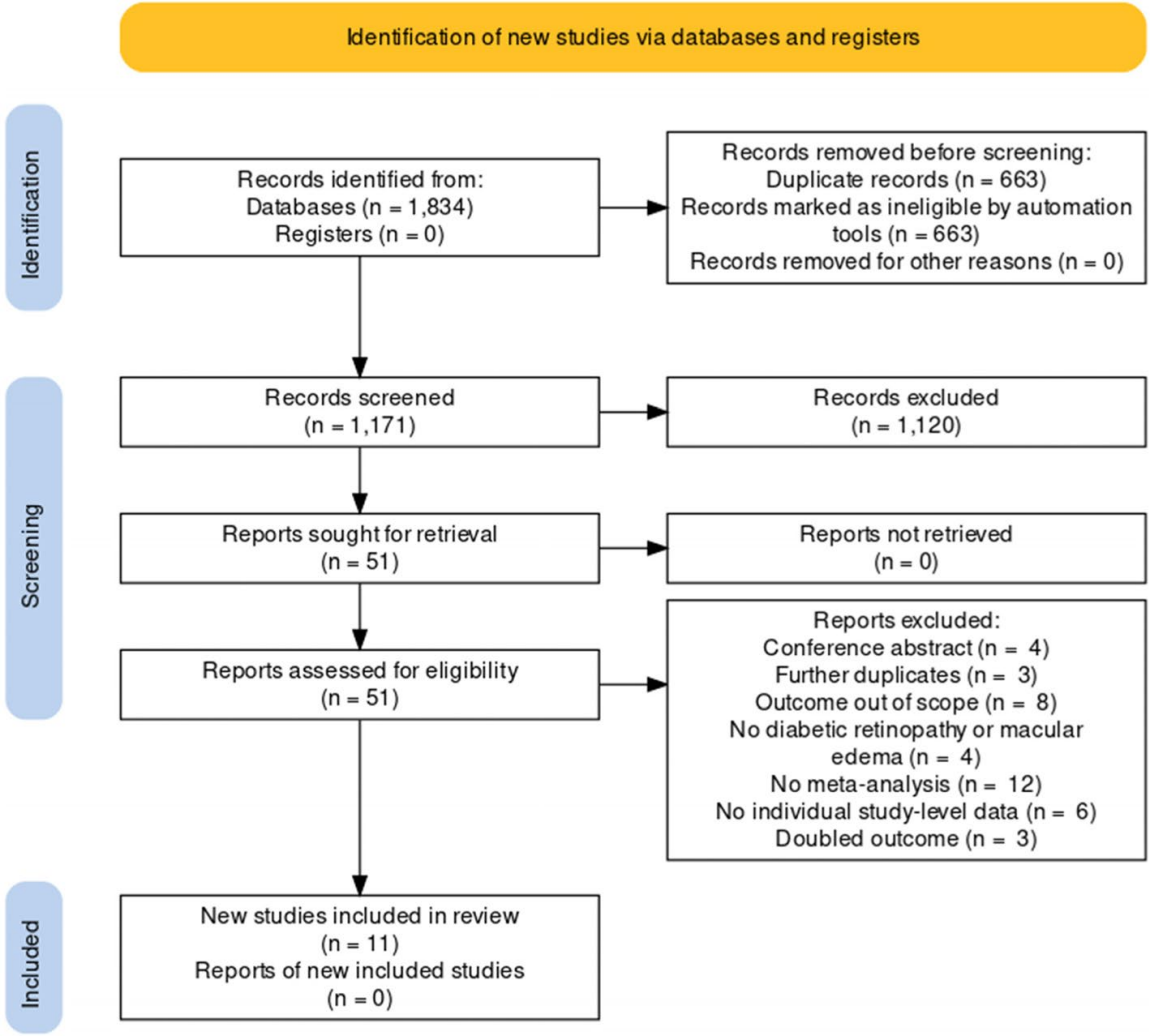
PRIMSA Flowchart of included studies and outcomes. PRISMA = Preferred Reporting Items for Systematic Reviews and Meta-analyses

The PRISMA flow chart is reported in Fig. 1. From 1,834 initial hits, 663 duplicates were automatically removed, leaving 1171 titles and abstracts to be assessed. After retrieving and assessing 51 full text articles following title and abstract review, 11 systematic reviews [5, 7, 8, 28–35] with meta-analyses were included with a total of 16 independent outcomes, with a total of 413,142 participants (median participants per outcome 7,266). Table 2 shows descriptive statistics of included studies and Supplementary Table 2 shows a list of full text studies that were assessed and excluded, with justifications.

**Table 2:** Descriptive characteristics of included studies and outcomes

Author	Type of outcome	Sub-type of outcome	Study type(s)	Total included studies	Total participants
Zhu et al. [28]	Mortality	All-cause mortality	Cohort	18	19,813
Xu et al. [5]		CVD mortality in all DR	Cohort	10	11,239
		CVD mortality in mild DR		4	7,361
		CVD mortality in severe DR		6	9,691
Zhu et al. [8]	Disease	Obstructive sleep apnoea	Case–control	6	1,092
Kjærsgaard et al. [35]		Primary open angle glaucoma	Cohort	2	241,037
			Cross-sectional	4	15,389
Shiferaw et al. [30]		Chronic kidney disease	Cohort	2	781
Song et al. [31]		Non-alcoholic fatty liver disease all DR	Cross-sectional	8	7,170
		Non-alcoholic fatty liver disease non-proliferative DR		3	3,977
		Non-alcoholic fatty liver disease proliferative DR		3	3,977
Wu and You [7]	Mental health	Depression (Type I)	Case–control	3	971
Zou et al. [29]		Depression (Type II)	Cross-sectional	9	31,766
Trott et al. [32]		Eating disorders	Cross-sectional and case–control	7	1,091
Chai et al. [33]		Dementia	Cohort	3	46,185
		Alzheimer’s	Cohort	2	7,754
Wu et al. [34]		Cognitive impairment	Cross-sectional	5	2,451
			Cohort	5	1,397

#### Meta-analysis

Ten outcomes yielded statistically nominal results, seven of which were graded as Grade II, with the remaining three outcomes being graded at grade IV (see Table 3). Five of these yielded low heterogeneity (I^2^ < 50%), three yielded moderate heterogeneity (I^2^ = 50–75%), with the remaining two outcomes yielding high heterogeneity (I^2^ =  > 75%). Five significant outcomes yielded a PI that excluded the null, five had evidence of small-study effects, while two had evidence of excess significance bias (see Table 3 for full details). Only one outcome (depression) yielded outcomes stratified by type of diabetes.

**Table 3:** Meta-analysis results

Arial	8	Study type(s)	Total included studies	Total participants	Effect size type	Effect size (95% CI)	p	I^2^	Small study effect	Excess significance bias	PI	Level of evidence
**Mortality**	All-cause mortality	Cohort	18	19,813	RR	2.37 (2.02–2.78)	< 1^–6^	42.31	Yes	No	1.50–3.73	Grade II
	CVD mortality in all DR		10	11,239	RR	1.83 (1.42–2.36)	< 1^–6^	76.28	No	Yes	0.81–4.13	Grade II
	CVD mortality in mild DR		4	7,361	RR	1.14 (0.81–1.58)	0.45	63.24	No	NS	0.30–4.29	NS
	CVD mortality in severe DR		6	9,691	RR	2.26 (1.31–3.91)	< 1^–6^	84.96	No	No	0.37–13.8	Grade II
**Disease**	Obstructive sleep apnoea	Case–control	6	1,092	OR	2.16 (1.35–3.44)	0.001	52.12	No	No	0.57–8.74	Grade II
	Chronic kidney disease	Cohort	2	781	OR	2.73 (0.37–19.95)	0.32	97.22	No	NS	NA	NS
	Non-alcoholic fatty liver disease all DR	Cross-sectional	8	7,170	OR	1.00 (0.48–2.10)	1.00	96.65	Yes	NS	0.07–14.9	NS
	Non-alcoholic fatty liver disease non-proliferative DR		3	3,977	OR	0.74 (0.37–1.50)	0.40	95.25	No	NS	0.00–5561.39	NS
	Non-alcoholic fatty liver disease proliferative DR		3	3,977	OR	0.96 (0.21–4.28)	0.96	96.53	No	NS	0.00–162,240,006.20	NS
	Primary open angle glaucoma	Cohort	2	241,037	OR	1.47 (0.57–3.77)	0.42	95.45	No	NS	NA	NS
		Cross-sectional	4	15,389	OR	1.01 (0.56–1.81)	0.98	84.32	No	NS	0.07–14.14	NS
**Mental Health**	Depression (Type I)^†^	Case–control	3	971	OR	2.19 (0.91–5.24)	0.08	84.99	Yes	NS	0.00–86,679.18	NS
	Depression (Type 2) ^†^	Cross-sectional	9	31,766	OR	1.62 (1.37–1.91)	< 1^–6^	43.36	No	Yes	1.09–2.41	Grade II
	Eating disorders	Cross-sectional	7	1,091	OR	2.81 (1.67–4.72)	< 1^–6^	35.21	No	No	0.82–9.63	Grade II
	Alzheimer’s	Cohort	2	46,185	OR	1.56 (1.16–2.08)	0.003	0.00	Yes	No	NA	Grade IV
	Dementia	Cohort	3	7,754	OR	1.46 (1.09–1.94)	0.01	73.64	No	No	0.05–39.66	Grade IV
	Cognitive impairment	Cross-sectional	5	2,451	OR	2.07 (1.11–3.88)	0.02	60.03	No	No	0.28–15.06	Grade IV
		Cohort	5	1,397	OR	2.71 (1.90–3.87)	< 1^–6^	0.02	Yes	No	1.90–3.87	Grade II

^†^ Depression was the only outcome that stratified by type of diabetes – all other outcomes were mixed

#### Risk of bias

All but one meta-analyses scored critically low as assessed by AMSTAR2. Primary reasons for this classification were not providing a list of excluded studies with justified exclusions, and not reporting that the review had a protocol that was established prior to the review (see Supplementary Table 3).

#### Mortality

**Fig. 2:**
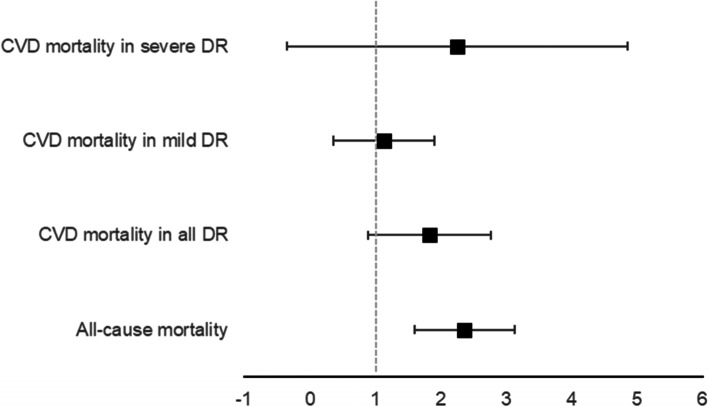
Pooled risk ratios showing the associations between mortality and diabetic retinopathy. DR = diabetic retinopathy

Four outcomes assessed associations between the risk of DR and mortality, including all-cause mortality, and cardiovascular mortality (in all DR, ‘mild’ DR, and ‘severe’ DR; ‘severe’ DR was defined as ‘proliferative DR, severe non-proliferative DR, sight-threatening DR, or any combination of these categories’). All-cause mortality (RR = 2.37 95%CI 2.02–2.78), cardiovascular mortality in all DR (RR = 1.83 95% CI 1.42–2.36) and ‘severe’ DR (RR = 2.26 95% CI 1.31–3.91) all yielded significant associations, whereas cardiovascular mortality was not significantly associated with ‘mild’ DR (RR = 1.14 95%CI 0.81–1.58). See Fig. 2 for a graphical representation.

#### Disease

**Fig. 3:**
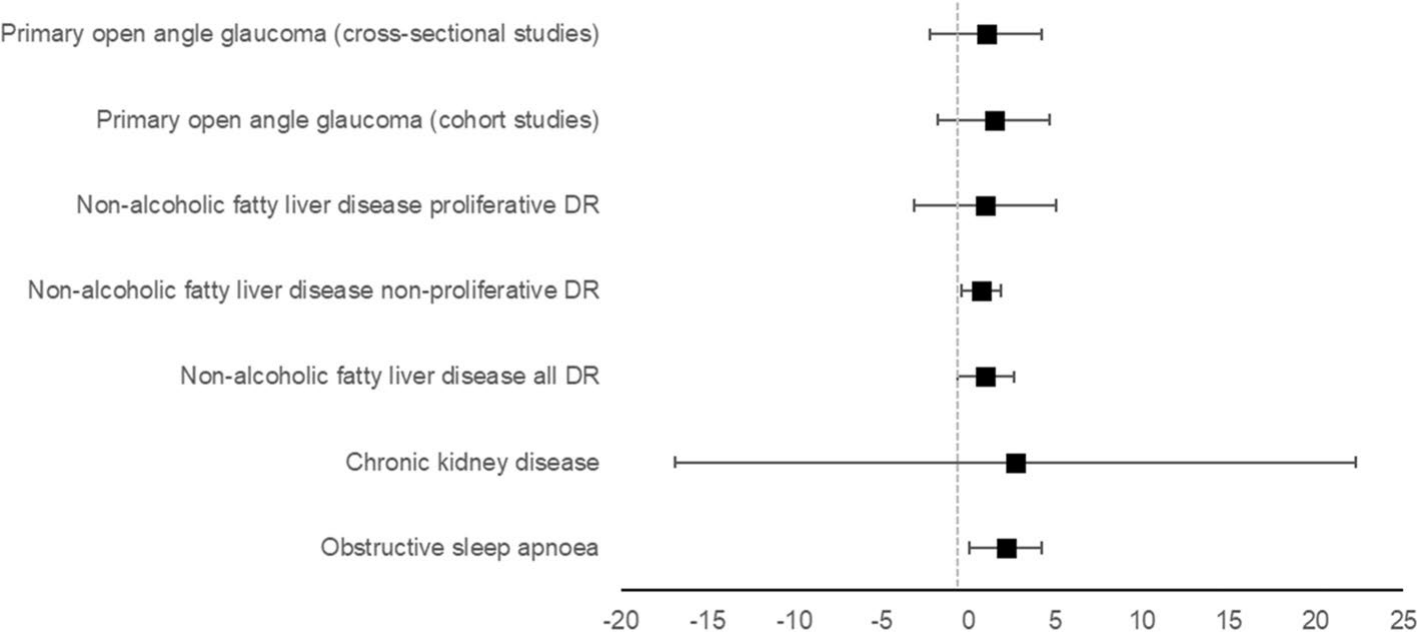
Pooled odds ratios showing the associations between disease outcomes and diabetic retinopathy. DR = diabetic retinopathy

Six outcomes assessed associations between DR risk and diseases, including chronic kidney disease (CKD), non-alcoholic fatty liver disease (NAFLD; three outcomes, all DR, non-proliferative DR, and proliferative DR), obstructive sleep apnoea (OSA), stroke, and primary open angle glaucoma (POAG). OSA and stroke were significantly associated with DR risk (OSA: OR = 2.16 95% CI 1.35–3.44; stroke: RR = 1.74 95% CI 1.35–2.24). All other outcomes yielded non-significant results (CKD: OR = 2.73 95%CI 0.37–19.95; NAFLD and all DR: OR = 1.00 95%CI 0.48–2.10; NAFLD non-proliferative DR: OR = 0.74 95% CI 0.37–1.50; NAFLD proliferative DR: OR = 0.96 95% CI = 0.21–4.28; POAG cohort studies OR = 1.47 95% CI 0.57–3.77; POAG cross-sectional studies OR 1.01 95% CI 0.56–1.81). See Fig. 3 for a graphical representation.

#### Mental health

**Fig. 4:**
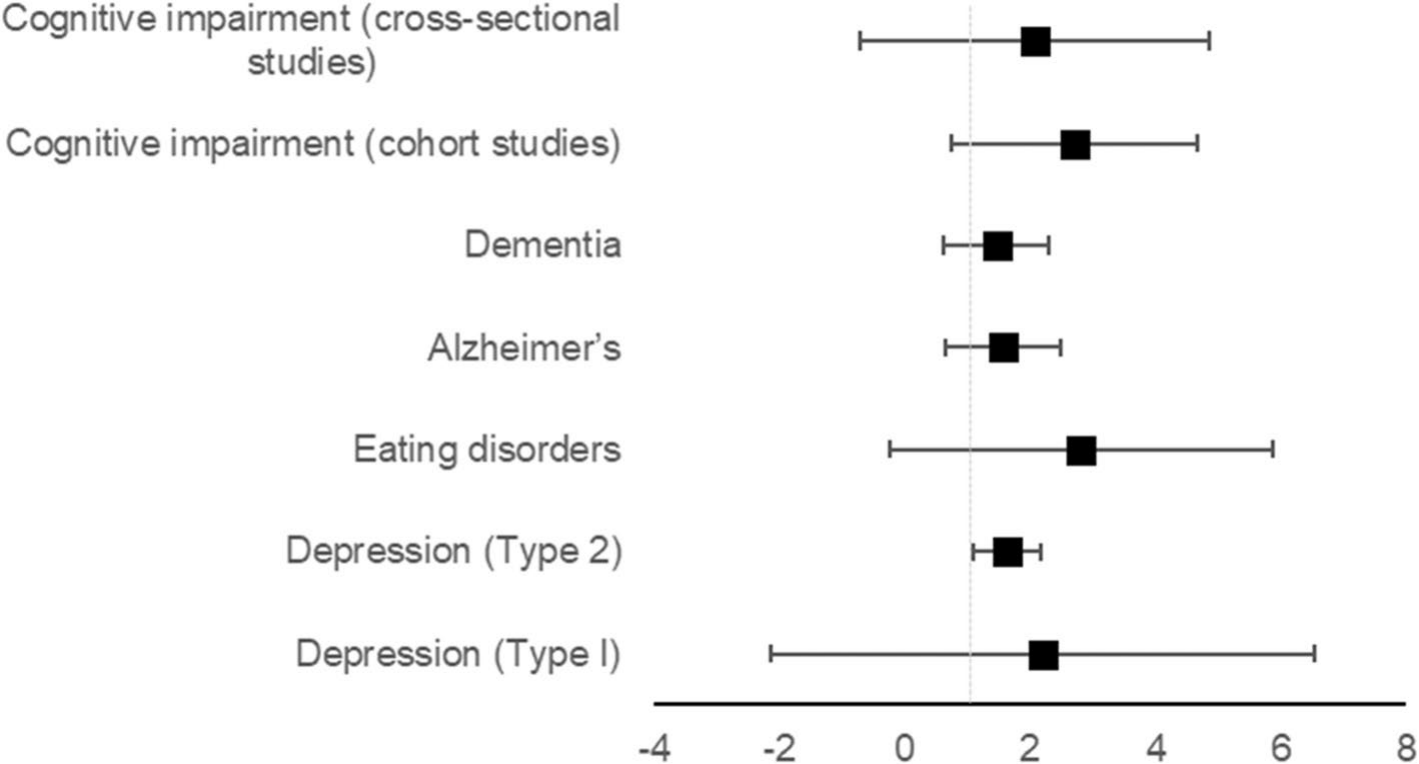
Pooled odds ratios showing the associations between mental health outcomes and diabetic retinopathy

Six outcomes examined associations between DR risk and mental health disorders, including depression (in type I and type II diabetes as stratified outcomes) eating disorders, Alzheimer’s disease, dementia, and cognitive impairment. Depression in participants with type II diabetes yielded significant associations (OR = 1.62 95%CI 1.37–1.91), whereas depression in participants with type I diabetes did not yield significant associations. Eating disorders (OR = 2.81 95%CI 1.67–4.72), Alzheimer’s disease (OR = 1.56 95%CI 1.16–2.08), dementia (OR = 1.46 95%CI 1.09–1.94), and cognitive impairment (in cross-sectional studies OR = 2.07 95%CI 1.11–3.88, and cohort studies OR = 2.71 95%CI 1.90–3.87) were all statistically significant. See Fig. 4 for a graphical representation.

## Discussion

This review of reviews, which included 11 studies spanning 16 independent outcomes, provides an overview of the current meta-analytic evidence of associations between DR, mortality, disease, and mental health conditions. Furthermore, this review provides a systematic evaluation of the epidemiological credibility of these studies. According to the GRADE criteria, seven significant outcomes yielded Grade II evidence, which signifies a high degree of confidence in the credibility of significant evidence. The remaining three significant associations were graded as Grade IV, which indicates a low degree of confidence.

### Mortality

Of the outcomes that examined DR and mortality, all but one (CV mortality and mild DR) yielded significant associations, all of which were large effect sizes and graded as Grade II (high degree of confidence). The risk of all-cause mortality appears to be more than double in people with DR compared to people with no evidence of DR. Furthermore, the risk of CV mortality was nearly double in people with DR compared to people without, with this risk increasing if a patient had ‘severe’ (defined as ‘proliferative DR, severe non-proliferative DR, sight-threatening DR, or any combination of these categories’) DR. The finding that the association between ‘mild’ DR and CV mortality is particularly interesting, indicating that the risk of mortality may increase as DR progresses into the sight-threatening stages [5]. Indeed, Miettinen and colleagues found in a large cohort study (with seven-year follow up) that only proliferative DR (sight threatening) was a significant risk factor of CV mortality [36]. These findings, however, need to be considered with caution – the mild DR analysis had fewer studies and participants than the other analyses, so these results could be due to smaller statistical power. These results do provide further evidence, however, that retarding the progression of DR (and, indeed, initial onset) is of paramount importance in people with diabetes.

### Disease

Of the outcomes that examined DR and disease, only obstructive sleep apnoea (OSA) was significantly associated with DR. OSA has been well-reported to have accompanying nocturnal decreases in oxygen saturation [37], leading to nocturnal hypoxia. In turn, the retina has been reported to be sensitive to hypoxia, and this chronic hypoxia could lead to several inflammatory and oxidative stress reactions [8], which could lead to endothelia dysfunction, and a subsequent increase in DR risk.

### Mental health

In this analysis, depression, the presence of eating disorders, and several types of cognitive impairment were all strongly associated with DR, with varying degrees of epidemiological credibility. Regarding depression, significant associations were only found between DR and depression in Type 2 diabetes, however this could be because of the limited statistical power of the type 1 analysis. Further research examining depression and DR in patients with type I diabetes is warranted. There are several potential mechanisms for this dependent on the temporal relationship. For example, the activation of sympathetic nervous system and increases in cortisol and catecholamine levels as a result of depression could cause changes in insulin resistance and glycaemic function, both of which can increase DR risk [29, 38, 39]. On the other hand, people with DR may experience depression because of fear of blindness, vision loss, and decreased quality of life [29].

This review found a strong significant association between eating disorders and DR risk. It is likely that the mechanism behind this risk is due to the frequent manipulation of insulin to achieve weight loss, leading to poor glycaemic control, which is an established risk factor for DR [40]. It is recommended that patients with diabetes be regularly monitored for eating disorder pathologies.

Regarding cognitive impairment, the results of this analysis highlight an already established link between cognitive decline and diabetes [41]. It has been reported that retinal and cerebral microvascular changes are similar, which could be a possible explanation for this association [42, 43]. It is difficult to determine, however, if the associations between DR and cognitive decline are independent of age. Indeed, it is well established that the risk of cognitive decline increases with age. Further meta-analyses examine this association should aim to only include studies that have adjusted for age to yield independent associations.

Umbrella reviews are a source of high-quality evidence synthesis, and this is the only review to our knowledge to assess meta-analytic associations between DR risk and mortality, disease, and mental health, whilst assessing their epidemiological credibility. The results of this study, however, should be considered within its limitations. Although heterogeneity was measured, the included studies had differing types of diabetes (with some studies not reported which type) and stages of DR, which could both be sources of heterogeneity. Further primary studies, and indeed reviews, should aim to stratify between type of DR and type of diabetes where possible. Moreover, none of the meta-analyses we encountered controlled their results for the duration of diabetes, which is a key indicator of DR. Furthermore, the findings are dependent on reported estimates that are selected from each primary study and how they are applied in each analysis [44]. Finally, almost all the included studies had critical reporting flaws that may preclude reproducibility (as seen in the AMSTAR2 ratings). It is important that future studies include critical quality indicators such as confirming protocols were pre-registered, or details about excluded studies, to minimise potential risk of bias, and increase transparency.

## Conclusion

The results of this study showed highly suggestive evidence of positive associations between DR and several types of mortality, including all-cause and CV mortality. OSA and several mental health conditions, including depression, eating disorders and different degrees of cognitive impairment were also associated with DR risk. Practitioners and public health professionals should take note of these when considering policies and treatments in order to reduce the risk of diabetic related blindness and other complications.

## Supplementary Information

**Additional file 1:** **Supplementary Table1. **Full search strategy.** Supplementary Table 2. **List of excluded full textstudies with reasons for exclusion. **Supplementary Table 3. **Fulldetails of AMSTAR2 results

## Data Availability

The datasets used and/or analysed during the current study are available from the corresponding author on reasonable request.

## Abbreviations

AMSTAR: Assessment of multiple systematic reviews tool
CI: Confidence interval
CKD: Chronic kidney disease
CV: Cardio-vascular
DME: Diabetic macular edema
DR: Diabetic retinopathy
E: Expected number of studies
ES: Effect size
MOOSE: Meta-analysis of Observational Studies in Epidemiology
NAFLD: Non-alcoholic fatty liver disease
OR: Odds ratio
OSA: Obstructive sleep apnoea
PRISMA: Preferred Reporting Items for Systematic Reviews and Meta-analyses
RR: Risk ratio
