# Supplementary material for: Associations between diabetic retinopathy, mortality, disease, and mental health: an umbrella review of observational meta-analyses
Source: BMC Endocr Disord. 2022 Dec 9;22:311. doi: 10.1186/s12902-022-01236-8 (PMC9733253; doi:10.1186/s12902-022-01236-8)
Supplement: Supplementary file 1 — Additional file 1: Supplementary Table1. Full search strategy. Supplementary Table 2. List of excluded full textstudies with reasons for exclusion. Supplementary Table 3. Fulldetails of AMSTAR2 results [file 12902_2022_1236_MOESM1_ESM.docx]

**Supplementary Table 1: Full search strategy**

| **Database** | **Search terms** | **Results** |
| --- | --- | --- |
| **Pubmed** | **(risk[Title/Abstract] OR risk factor[Title/Abstract]) AND (diabetic retinopathy[Title/Abstract] OR diabetic macular edema[Title/Abstract] OR diabetic macular oedema[Title/Abstract] OR proliferative diabetic retinopathy[Title/Abstract] OR proliferative retinopathy[Title/Abstract] OR sight threatening retinopathy[Title/Abstract] OR retinopathy[Title/Abstract]) AND (meta anal*[Title/Abstract] OR meta-anal*[Title/Abstract] OR systematic review[Title/Abstract])** | **723** |
| **Embase** | **(risk OR ‘risk factor’) AND (‘diabetic retinopathy’ OR ‘diabetic macular edema’ OR ‘diabetic macular oedema’ OR ‘proliferative diabetic retinopathy’ OR ‘proliferative retinopathy’ OR ‘sight threatening retinopathy’ OR retinopathy) AND (‘meta anal*’ or ‘meta-anal*’ or ‘systematic review’).ti.ab** | **866** |
| **Cinahl (via EMBASE)** | **(risk OR risk factor) AND (diabetic retinopathy OR diabetic macular edema OR diabetic macular oedema OR proliferative diabetic retinopathy OR proliferative retinopathy OR sight threatening retinopathy OR retinopathy) AND (meta anal* or meta-anal* or systematic review)** | **245** |

**Supplementary Table 2: List of excluded full text studies with reasons for exclusion**

| **Authors** | **Title of paper** | **Reason for exclusion** |
| --- | --- | --- |
| Alvarenga et al. | Association Between Diabetic Retinopathy and Periodontitis-A Systematic Review. | No meta-analysis |
| Hu et al. | Association of Diabetic Retinopathy With Stroke: A Systematic Review and Meta-Analysis. | No data on disease |
| de Groot et al. | Association of depression and diabetes complications: a meta-analysis. | No individual study data |
| Xie et al. | Association of Diabetic Macular Edema and Proliferative Diabetic Retinopathy With Cardiovascular Disease: A Systematic Review and Meta-analysis. | No individual study data |
| Wang et al. | Association of Helicobacter pylori infection with diabetic complications: a meta-analysis. | No individual study data |
| Isidro and Ruano | Bone disease in diabetes | No meta-analysis |
| Naskar et al. | Depression in diabetes mellitus-A comprehensive systematic review of literature from an Indian perspective | No meta-analysis |
| Chai et al. | Depression and Retinopathy in Patients With Type 2 Diabetes Mellitus: A Meta-Analysis. | Doubled data |
| Stana Serban et al. | Diabetic Retinopathy in Patients With Diabetic Foot Ulcer: A Systematic Review | No meta-analysis |
| Xu et al. | Diabetic retinopathy predicts cardiovascular mortality in diabetes: a meta-analysis | Duplicate |
| Vora | Diabetic retinopathy, cardiovascular events and mortality ... revisited. | No meta-analysis |
| Das et al. | Dyslipidemia and Diabetic Macular Edema: A Systematic Review and Meta-Analysis | Modifiable risk factor |
| Leong et al. | Effect of obstructive sleep apnoea on diabetic retinopathy and maculopathy: A systematic review and meta-analysis | No disease - meta-analysis is only for O2 saturation |
| Jonas and Sabanayagam | Epidemiology and Risk Factors for Diabetic Retinopathy | No meta-analysis |
| Lanting et al. | Ethnic differences in mortality, end-stage complications, and quality of care among diabetic patients: a review. | No meta-analysis |
| Sanal et al. | Factors responsible for poor control of type 2 diabetes mellitus - A systematic review and meta analysis | No DME or DR |
| Zhang et al. | Hypothyroidism and Adverse Endpoints in Diabetic Patients: A Systematic Review and Meta-Analysis | Duplicate |
| Xie et al. | Major risk factors of diabetic retinopathy and their relative importance: A meta analysis pooling data from individual subjects in 13 studies | Conference abstract |
| Hu et al. | Meta-analysis of the relationship between sleep duration and the risk of diabetic retinopathy | Not a disease |
| Pal et al. | Mild cognitive impairment and progression to dementia in people with diabetes, prediabetes and metabolic syndrome: a systematic review and meta-analysis | No DME or DR |
| Zhu et al. | Prediction of risk of diabetic retinopathy for all-cause mortality, stroke and heart failure Evidence from epidemiological observational studies | Duplicate |
| Mohammadi et al. | Prevalence and major causes of visual impairment in Iranian adults: A systematic review | Outcomes irrelevant |
| Song et al. | Prevalence, risk factors and burden of diabetic retinopathy in China: a systematic review and meta-analysis. | No individual study data |
| Virk et al. | Prevention of retinopathy in type 1 diabetes: A systematic review and meta-analysis | Conference abstract |
| Pederson et al. | Relationship between Diabetic Retinopathy and Systemic Neurodegenerative Diseases: A Systematic Review and Meta-analysis. | Doubled outcome |
| Dumitrascu et al. | Retinal Microvascular Abnormalities as Surrogate Markers of Cerebrovascular Ischemic Disease: A Meta-Analysis. | Retinopathy only - not specifically DR |
| Allon et al. | Retinal Microvascular Signs as Screening and Prognostic Factors for Cardiac Disease: A Systematic Review of Current Evidence | No meta-analysis |
| Swanson | Retinopathy screening in individuals with type 2 diabetes: Who, how, how often, and at what cost - An epidemiologic review | No meta-analysis |
| Fakkel et al. | Risk Factors for Developing Diabetic Peripheral Neuropathy: a Meta-analysis | No individual study data |
| Rossboth et al. | Risk factors for diabetic foot complications in type 2 diabetes-A systematic review | No meta-analysis |
| Kaminski et al. | Risk factors for foot ulceration and lower extremity amputation in adults with end-stage renal disease on dialysis: A systematic review and meta-analysis | No individual study data |
| Zhou and Yang | Risk of diabetic retinopathy from epidemiological observational studies | No full text available |
| Martinez Ceron et al.. | Sleep apnea-hypopnea syndrome and type 2 diabetes. A reciprocal relationship? | No meta-analysis |
| Drinkwater et al. | The relationship between carotid disease and retinopathy in diabetes: a systematic review. | No meta-analysis |
| Ikram et al. | Vision-threatening diabetic retinopathy and incident cardiovascular disease: A systematic review and meta-analysis | Conference abstract |
| Li et al. | Correlations among Diabetic Microvascular Complications: A Systematic Review and Meta-analysis | Diabetic complication |
| Huang et al. | Risk factors for the recurrence of diabetic foot ulcers among diabetic patients: a meta-analysis | Diabetic complication |
| Liu et al. | The risk factors for diabetic peripheral neuropathy: A meta-analysis | Diabetic complication |
| Zou et al. Zhongyu; Lu, Jie; Sun, Weihong | Visual Impairment, Eye Diseases, and Dementia Risk: A Systematic Review and Meta-Analysis. | Doubled outcome |
| Wu et al. | Visual Impairment, Major Eye Diseases, and Mortality in a Multi-Ethnic Asian Population and a Meta-analysis of Prospective Studies. | Not a disease |

**Supplementary Table 3: Full details of AMSTAR2 results**

| Author of Meta-Analysis | Year of Meta-Analysis | 1 | 2 | 3 | 4 | 5 | 6 | 7 | 8 | 9 | 10 | 11 | 12 | 13 | 14 | 15 | 16 | AMSTAR 2 Rating |
| --- | --- | --- | --- | --- | --- | --- | --- | --- | --- | --- | --- | --- | --- | --- | --- | --- | --- | --- |
| Chai et al. | 2022 | Yes | No | No | Partial yes | Yes | Yes | No | Partial yes | No | No | Yes | No | No | Yes | No | Yes | Critically low |
| Kjærsgaard et al | 2022 | Yes | Yes | No | Partial yes | No | Yes | No | Partial yes | Yes | No | Yes | Yes | No | Yes | Yes | Yes | Critically low |
| Song et al. | 2021 | Yes | No | No | Partial yes | Yes | Yes | No | Partial yes | Yes | No | Yes | Yes | Yes | Yes | Yes | Yes | Critically low |
| Shiferaw et al. | 2020 | Yes | Yes | No | Partial yes | Yes | Yes | No | Partial yes | Yes | No | Yes | No | No | No | Yes | Yes | Critically low |
| Trott et al. | 2022 | Yes | Yes | No | Partial yes | Yes | Yes | Yes | Yes | Yes | No | Yes | Yes | Yes | Yes | Yes | Yes | Moderate |
| Zou et al. | 2021 | Yes | No | No | Partial yes | Yes | No | No | Partial yes | No | No | Yes | No | No | No | Yes | Yes | Critically low |
| Xu et al. | 2020 | Yes | Yes | No | Partial yes | Yes | No | No | Yes | No | No | Yes | No | No | No | No | Yes | Critically low |
| Zhu et al. | 2017 | No | No | No | No | Yes | Yes | No | No | No | No | Yes | No | No | No | Yes | Yes | Critically low |
| Wu and You | 2018 | Yes | No | No | No | Yes | Yes | No | No | Yes | No | Yes | No | No | Yes | No | No | Critically low |
| Wu et al. | 2022 | Yes | No | No | Partial yes | Yes | Yes | No | Yes | Yes | No | Yes | Yes | Yes | Yes | Yes | Yes | Critically low |

AMSTAR2 Questions: Q1: Did the research questions and inclusion criteria for the review include the components of PICO?; Q2: Did the report of the review contain an explicit statement that the review methods were established prior to the conduct of the review and did the report justify any significant deviations from the protocol?; Q3: Did the review authors explain their selection of the study designs for inclusion in the review?; Q4: Did the review authors use a comprehensive literature search strategy?; Q5: Did the review authors perform study selection in duplicate?; Q6: Did the review authors perform data extraction in duplicate?; Q7: Did the review authors provide a list of excluded studies and justify the exclusions?; Q8: Did the review authors describe the included studies in adequate detail?; Q9: Did the review authors use a satisfactory technique for assessing the risk of bias (RoB) in individual studies that were included in the review?; Q10: Did the review authors report on the sources of funding for the studies included in the review?; Q11: If meta-analysis was performed did the review authors use appropriate methods for statistical combination of results?; Q12: If meta-analysis was performed, did the review authors assess the potential impact of RoB in individual studies on the results of the meta-analysis or other evidence synthesis?; Q13: Did the review authors account for RoB in individual studies when interpreting/ discussing the results of the review?; Q14: Did the review authors provide a satisfactory explanation for, and discussion of, any heterogeneity observed in the results of the review?; Q15: If they performed quantitative synthesis did the review authors carry out an adequate investigation of publication bias (small study bias) and discuss its likely impact on the results of the review?; Q16: Did the review authors report any potential sources of conflict of interest, including any funding they received for conducting the review?
